# Study of Silicon Nitride Inner Spacer Formation in Process of Gate-all-around Nano-Transistors

**DOI:** 10.3390/nano10040793

**Published:** 2020-04-20

**Authors:** Junjie Li, Yongliang Li, Na Zhou, Wenjuan Xiong, Guilei Wang, Qingzhu Zhang, Anyan Du, Jianfeng Gao, Zhenzhen Kong, Hongxiao Lin, Jinjuan Xiang, Chen Li, Xiaogen Yin, Xiaolei Wang, Hong Yang, Xueli Ma, Jianghao Han, Jing Zhang, Tairan Hu, Zhe Cao, Tao Yang, Junfeng Li, Huaxiang Yin, Huilong Zhu, Jun Luo, Wenwu Wang, Henry H. Radamson

**Affiliations:** 1 Key Laboratory of Microelectronics Devices & Integrated Technology, Institute of Microelectronics, Chinese Academy of Sciences, Beijing 100029, China; liyongliang@ime.ac.cn (Y.L.); zhouna@ime.ac.cn (N.Z.); xiongwenjuan@ime.ac.cn (W.X.); zhangqingzhu@ime.ac.cn (Q.Z.); duanyan@ime.ac.cn (A.D.); gaojianfeng@ime.ac.cn (J.G.); kongzhenzhen@ime.ac.cn (Z.K.); linhongxiao@ime.ac.cn (H.L.); xiangjinjuan@ime.ac.cn (J.X.); lichen2017@ime.ac.cn (C.L.); yinxiaogen@ime.ac.cn (X.Y.); wangxiaolei@ime.ac.cn (X.W.); yanghong@ime.ac.cn (H.Y.); maxueli@ime.ac.cn (X.M.); hanjianghao@ime.ac.cn (J.H.); tyang@ime.ac.cn (T.Y.); lijunfeng@ime.ac.cn (J.L.); yinhuaxiang@ime.ac.cn (H.Y.); zhuhuilong@ime.ac.cn (H.Z.); rad@ime.ac.cn (H.H.R.); 2 Microelectronics Institute, University of Chinese Academy of Sciences, Beijing 100049, China; 3 State Key Laboratory of Advanced Materials for Smart Sensing, General Research Institute for Nonferrous Metals, Beijing 100088, China; 4 College of Electronic and Information Engineering, North China University of Technology, Beijing 100144, China; zhangj@ncut.edu.cn (J.Z.); tairanhu1@gmail.com (T.H.); chrisaigakki@gmail.com (Z.C.); 5 Department of Electronics Design, Mid Sweden University, Holmgatan 10, 85170 Sundsvall, Sweden

**Keywords:** inner spacer, gate-all-around (GAA), nanowire, nanosheet, field effect transistor, nanostructure manufacture, high anisotropy, high etch selectivity

## Abstract

Stacked SiGe/Si structures are widely used as the units for gate-all-around nanowire transistors (GAA NWTs) which are a promising candidate beyond fin field effective transistors (FinFETs) technologies in near future. These structures deal with a several challenges brought by the shrinking of device dimensions. The preparation of inner spacers is one of the most critical processes for GAA nano-scale transistors. This study focuses on two key processes: inner spacer film conformal deposition and accurate etching. The results show that low pressure chemical vapor deposition (LPCVD) silicon nitride has a good film filling effect; a precise and controllable silicon nitride inner spacer structure is prepared by using an inductively coupled plasma (ICP) tool and a new gas mixtures of CH_2_F_2_/CH_4_/O_2_/Ar. Silicon nitride inner spacer etch has a high etch selectivity ratio, exceeding 100:1 to Si and more than 30:1 to SiO_2_. High anisotropy with an excellent vertical/lateral etch ratio exceeding 80:1 is successfully demonstrated. It also provides a solution to the key process challenges of nano-transistors beyond 5 nm node.

## 1. Introduction

In order to overcome challenges such as short channel effect brought by scaling down metal-oxide-semiconductor field-effect transistors (MOSFETs), many new devices e.g., fin field effective transistors( FinFETs), tunneling field-effect transistors (TFETs), ultra-thin-body transistors (ULBTs) and gate-all-around nanowire transistors (GAA-NWTs) have been developed [1,2,3]. However, among this group, nanowire transistors are considered to be the most competitive devices in the future [4,5]. In general, there are two main solutions for manufacturing the (horizontal or vertical) nanowires: one is a bottom-up bulk silicon-based process [6,7]; the other is a top-down fabrication approach using SiGe/Si stacks and selectively etch SiGe as channel material. The latter is more likely to become the mainstream solution for the technology generation below 5nm technology node, because the device’s process is very similar to conventional FinFET process flow [8,9].

**Figure 1:**
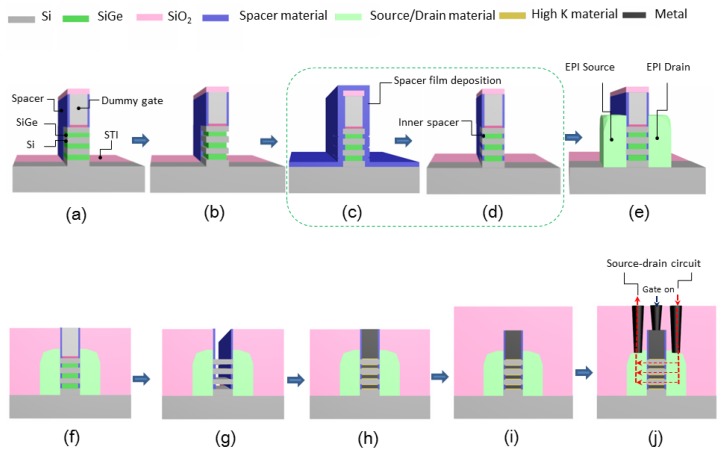
Process flow of nanowires with inner spacer: (**a**) source/drain Fin recess for opening active area; (**b**) SiGe cavity etching for defining the growth position and size of the inner spacer; (**c**) inner spacer film deposition; (**d**) controlled etching of spacer film and formation of inner spacer; (**e**) source and drain epitaxial growth; (**f**) dielectric deposition and planarization; (**g**) dummy gate removal and silicon nanowires formation; (**h**) filling and planarization of high-K metal gates; (**i**) interlayer dielectric deposition; (**j**) metal contact plug and current direction when device is on.

Inner spacer was designed to reduce the parasitic capacitance between the gate and source/drain in stacked SiGe/Si structure GAA-NWTs [10,11]. The main process flow of GAA devices including the inner spacer process module is shown in Figure 1. The steps with challenges are SiGe cavity etching step and inner spacer formation with precise profile control and no damaging for the nanowires [12,13]. There have been several research reports on SiGe cavity etching [14,15,16], but a systematic investigation on the formation of inner spacers has still not been investigated.

**Figure 2:**
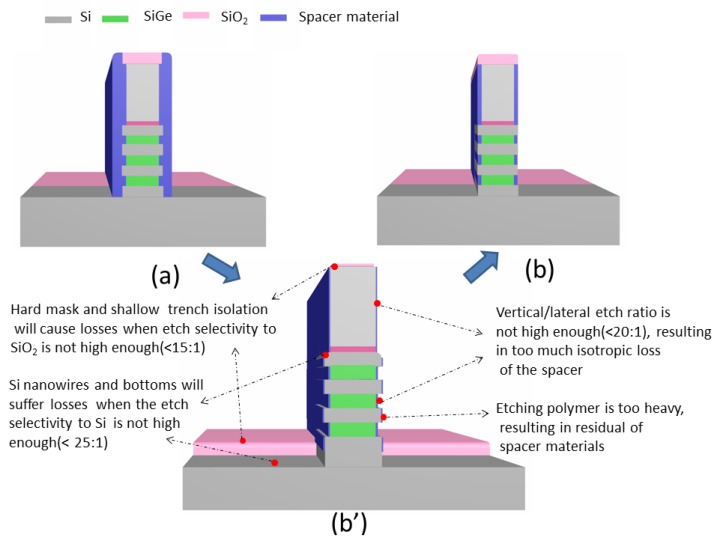
Spacer morphology and inner spacer process challenges: (**a**) conventional spacer; (**b**) inner spacer; (**b’**) the process challenges need to be overcome from conventional spacer to inner spacer.

In comparison with the conventional spacer process, the inner one has more process challenges. As shown in Figure 2a, the requirement of conventional spacer etching is that the spacer material on the top and bottom sides of the gate need to be etched completely, leaving the spacer material on the sidewall. Therefore, this process does not require a high etching selectivity and anisotropy. However, the higher anisotropy and etching selectivity are required to meet the requirements without any causing device failures in process of inner spacers formation [12,17], as shown in Figure 2b,b’.

SiNx with atomic ratio for Si to N of 3:4 is commonly used as spacer material [18,19]. For the etching of SiN, CF_4_/O_2_/N_2,_ CF_4_/CH_4_, SF_6_/CH_4_ and NF_3_/CH_4_ were used for conventional plasma etching in the early period, but the selectivity etching of SiN to SiO_2_ and Si are not high when the polymer is produced properly [20]. Using neutral beam reaction system, the SiN etch selectivities of 18.6 to SiO_2_ and 6.2 to Si can be achieved [21]. Later, BEE Kastenmeier et al. found that the use of microwave remote plasma can significantly improve the etch selectivity of SiN to Si and SiO_2_, and the ratio can reach to 70:1 [22]. However, because of the characteristics of partial isotropic etching, remote plasma is more suitable for SiN sacrificial layer removal than SiN spacer etching.

In recent years, quasi-atomic layer etching (QALE) of SiN has emerged [23], mainly using a two-step alternating method. At first, the surface is modified by using hydrogen ion implantation or plasma treatment, and then etching this surface with F-based process gas. Then, these two processes are alternately performed to achieve the purpose of quantitative etching. This method takes into account both the etching selection ratio and the anisotropic, but the quasi-atomic layer etching equipment is complex and the productivity is low, and no related public report shows that it has been used in the GAA nanowire inner spacer module [24,25,26,27].

In this work, a novel gas mixture of CH_2_F_2_/CH_4_/O_2_/Ar was used for etching the SiN inner spacer in GAA transistors in a conventional inductively coupled plasma (ICP) tool. This method avoids using specially designed hardware equipment and offers higher process efficiency than solutions such as ALE. Moreover, the conformal deposition and selective anisotropic etching process of inner spacer were also systematically studied. Firstly, the influence of the film deposition process on the filling effect of the inner spacer is discussed by comparing the plasma enhanced chemical vapor deposition (PECVD) and low-pressure chemical vapor deposition (LPCVD) methods. Later, the effects of main etching process parameters (CH_4_ flow, O_2_ flow and pressure) on the etch selectivity, anisotropy and etching morphology are investigated. Finally, high-resolution scanning electron microscope (HRSEM) (Hitachi Inc, Tokyo, Japan), high-resolution transmission electron microscope (HRTEM) (Thermo Fisher scientific Inc., Waltham, MA, USA)and energy dispersive spectrometer (EDS) (Thermo Fisher scientific Inc., Waltham, MA, USA) were used to analyze the microscopic details of the filling and etching of the inner spacer.

## 2. Materials and Methods 

**Figure 3:**
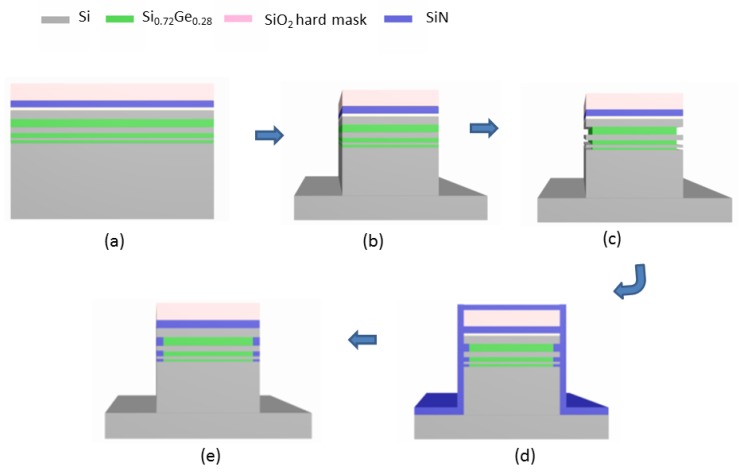
Schematic view of process flow to form the inner spacer: (**a**) Si_0.72_Ge_0.28_/Si multilayer structure(MLs )and hard mask growth, (**b**) lithographic patterning and plasma anisotropic etching, (**c**) Si_0.72_Ge_0.28_ isotropic selective etching; (**d**) SiN thin film deposition and filling and (**e**) SiN inner spacer anisotropic selective etching.

All the materials in this work were performed on 8-inch (100) silicon wafers. The experimental process and method are shown in Figure 3:

Step 1: Three cycles of SiGe/Si multilayers were grown by using reduced pressure chemical vapor deposition (RPCVD) technique [28,29], and then an oxide-nitride-oxide (ONO) hard mask were grown on the top silicon by applying plasma enhanced chemical vapor deposition (PECVD). In order to examine the film filling and etching performance of inner spacer in detail, Si_0.72_Ge_0.28_ stacks with different thicknesses are designed.

Step 2: 3 µm equally spaced line arrays were patterned, and the whole structure including the hard mask and Si_0.72_Ge_0.28_/Si stack are vertically etched to the substrate silicon by using the plasma etching. Finally, oxygen plasma is used to remove the photoresist [15].

Step 3: In the ICP etching tool, the Si_0.72_Ge_0.28_ layers were selectively etched by CF_4_/O_2_/He gas without any bias power, to obtain the lateral depth of 50–70 nm [15]. 

Step 4: For the cavity formed in the step 3, PECVD (AMAT Producer 200 mm(Applied Materials Inc., Santa Clara, CA, USA )) and low-pressure chemical vapor deposition (LPCVD) (AMAT Centura 200 (Applied Materials Inc., Santa Clara, CA, USA )) equipments were used to grow 40 nm SiN in the filling experiments. The growth temperature of PECVD was at 400 °C, while for LPCVD was at 750 °C. The growth temperatures at these steps were kept below 800 °C, in order to avoid the interdiffusion at the interfaces between Si/SiGe [30].

Step 5: Finally, the prepared samples were etched in an ICP tool (TCP 9400DFM (Lam Research Inc., Fremont, CA, USA)), where a gas mixture of CH_2_F_2_/O_2_/CH_4_/Ar and a chuck temperature of 80 °C were used. The research focuses on the effects of etching process parameters on selection ratio, anisotropy (vertical/lateral etch ratio) and etch morphology.

## 3. Results and Discussion

### 3.1. Effect of Thin Film Process on Gap Filling

**Figure 4:**
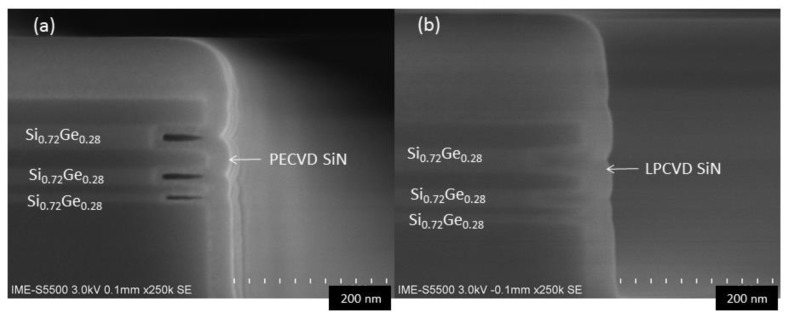
SEM images of the SiN inner spacer filling by using: (**a**) plasma enhanced chemical vapor deposition (PECVD) and (**b**) low-pressure chemical vapor deposition (LPCVD).

Inner spacers require thin films to grow uniformly to the sidewalls in the cavity; therefore, a good gap filling ability is required for the film growth. Atomic layer deposition (ALD) high-K materials (such as HfO_2_, ZrO_2_) have very good filling properties, but these materials increase parasitic capacitance and are detrimental to device performance, therefore these materials are not suitable choices. However, the fabrication of the GAA Si-Ge based nanowire devices using FinFET process flow usually requires special nanowire/sheet selective etching and surface processing including interfacial layer removal, diameter reduction and rounding in the advanced replacement metal gate (RMG) module [31,32]. These processes may bring great fabrication challenges for conventional low K material spacer. Therefore, the highly resistant and density SiN (K value is ~7) is still the best choice for spacer materials [12]. Meanwhile, for structures with lateral openings, then high-density plasma chemical vapor deposition (HDPCVD), which has good filling performance in the vertical hole structure becomes theoretically ineffective [33] and the damage caused by high-density plasma is inevitable. In this study, the filling effects of two conventional SiN thin film deposition techniques, PECVD and LPCVD were compared. The results are shown in Figure 4: The filling effect of LPCVD silicon nitride is significantly better than that of PECVD. The HRSEM micrographs reveals that, there are obvious holes in the PECVD grown layers, and the ratio of the voids in the original cavity: SiGe layers 10 nm > 20 nm > 30 nm. Meanwhile, silicon nitride which was grown by LPCVD did not show any holes in SiGe cavity with depths of 10 nm, 20 nm or 30 nm, showing LPCVD as a better conformal growth. More importantly, LPCVD SiN has better corrosion resistance than PECVD to facilitate subsequent process integration. This good property of using LPCVD is due to lower chamber pressure and higher temperature, which results in slower growth rate, better conformal coverage and higher density [34]. More detailed results on LPCVD silicon nitride will be discussed in the subsequent TEM and EDS characterizations in Section 3.5.

### 3.2. Effect of CH_4_ Flow on Inner Spacer Etching

**Figure 5:**
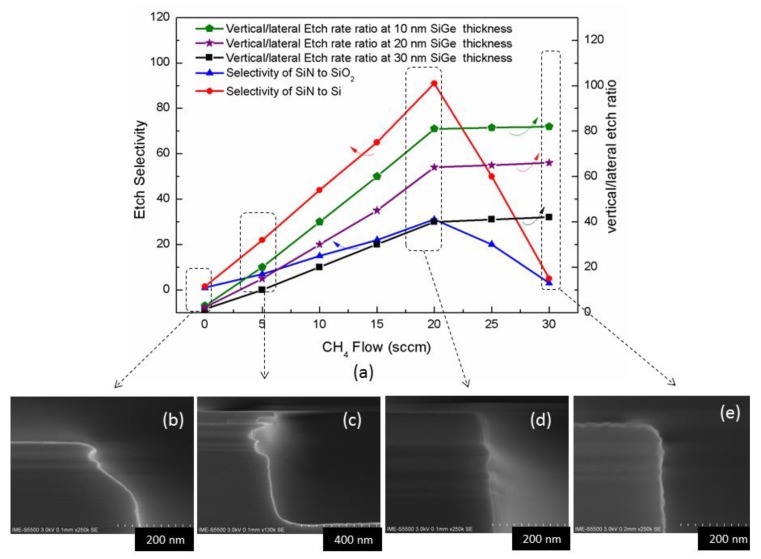
Impact of CH_4_ flow on etching: (**a**) the dependence of etch selectivity and vertical/lateral etch ratio on CH_4_ flow; (**b**) etching profile without CH_4_; (**c**) etching profile of 5 sccm CH_4_ flow; (**d**) etching profile of 20 sccm CH_4_ flow; (**e**) etching profile of 30 sccm CH_4_ flow;

More investigations were carried out to find the impact of CH_4_ gas flow on the etching profile while all other parameters were kept constant. The CH_4_ flow rate was varied by applying the following conditions: 80 mTorr/source RF 250 W/bias RF 35 W/*x* sccm CH_4_/25 sccm CH_2_F_2_/20 sccm O_2_/50 sccm Ar. The results are shown in Figure 5a. When there is no CH_4_ gas in the reaction chamber, it is found that the silicon nitride on sidewall is etched completely, while the top hard mask is totally consumed and the Si/SiGe stack is seriously damaged (Figure 5b), due to the etch selectivity as well as anisotropy are poor in absence of CH_4_. Meanwhile, as CH_4_ is inserted in the chamber, then the anisotropy during the etching is significantly improved. This is linked to the C-based polymer produced by CH_4_ passivates the sidewalls and increases the vertical/lateral etch ratio. This refers to the fact that CH_4_ reaction system has the highest C/F ratio compared to other CH_x_F_y_ mixed gases. The vertical/lateral etch ratio increases with the decrease in the thickness of the SiGe layer, because the aspect ratio dependent etch rate (ARDE) effect leads to a lower lateral etch rate for small-sized trenches under the same conditions [35].

At the same time, the increasing flow rate of CH_4_ improves the etch selectivity of silicon nitride to Si and SiO_2_. The mechanism is explained as the C and H elements in CH_4_ combine with the N in silicon nitride to form volatile HCN, which promotes the silicon nitride etching reaction. In addition, the selectivity ratio of silicon nitride to Si is higher than that of SiO_2_ because of C in CH_4_ which combines F in CH_2_F_2_ and O in SiO_2_ to form volatile COF_2_ does not affect the Si in similar way. Then, this trend reaches a peak when the flow of CH_4_ was 20 sccm, and the profile is relatively well controlled (Figure 5d). Finally, by continuing to increase CH_4_, the contribution to the anisotropy becomes small and the contribution of sidewall passivation reaches to a saturation level. The increase of CH_4_ flow greatly reduces the proportion of the F-based source gas CH_2_F_2_, then the silicon nitride etching rate decreases since the Si in silicon nitride needs to be combined with more F atoms to generate volatile SiF_4_. As the etch selectivity decreases, the hard mask material on the top of the structure is significantly consumed and the roughness of the sidewalls becomes worse (Figure 5e).

### 3.3. Effect of O_2_ Flow on Inner Spacer Etching

**Figure 6:**
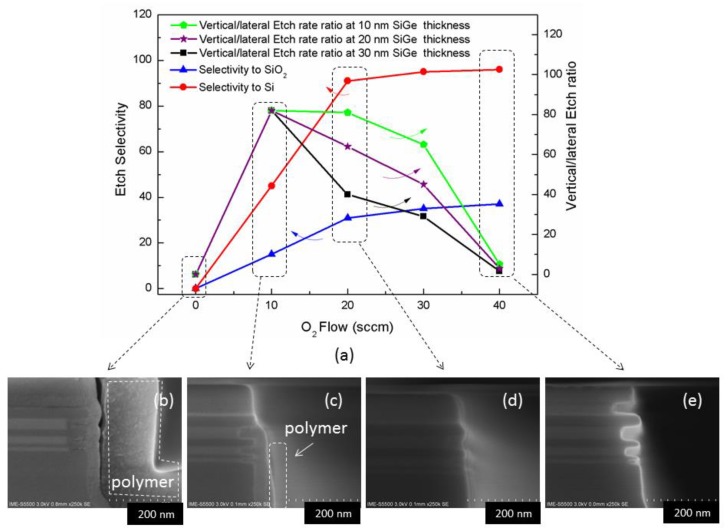
Effect of O_2_ flow on etching: (**a**) the dependence of etch selectivity and vertical/lateral etch ratio on O_2_ flow; (**b**) etching profile without O_2_; (**c**) etching profile of 10 sccm O_2_ flow; (**d**) etching profile of 20 sccm O_2_ flow; (**e**) etching profile of 30 sccm O_2_ flow.

In these experiments, O_2_ flow was changed while the other process parameters were unchanged as following: 80 mTorr/source RF 250 W/bias RF 35 W/20 sccm CH_4_/25 sccm CH_2_F_2_/*x* sccm O_2_/50 scccm Ar. The results are shown in Figure 6a. When there is no oxygen, a polymer deposition occurs on the surface of the structure (Figure 6b). When the O_2_ flow is increased to 10 sccm, the deposition is reduced, but the deposition is still visible on the side walls (Figure 6c). The polymer produced in the reaction is too heavy and the sidewalls are difficult to be completely etched. The mechanism can be explained that in the absence of O, CH_2_F_2_ and CH_4_ can easily form CH*_x_*F*_y_* polymers. Then, introducing O_2_ will generate volatile CO which reduces the amount of polymer formation but allowing other elements such as F to be released for etching silicon nitride [35]. When the amount of O_2_ reaches to 20 sccm, an equilibrium point is obtained. The etch selectivity and anisotropy are improved and as a result the etch profile becomes better (Figure 6d). As the amount of O_2_ continues to increase, the etching appears isotropic (Figure 6e is a typical SiN isotropic etching). The reason for this outcome is that O_2_ excessively consumes C in the reaction gas to form CO volatiles, which leads to a serious shortage of the CH*_x_*F*_y_* amount which is necessary for the protection of the side walls.

### 3.4. Effect of Pressure on Inner Spacer Etching

**Figure 7:**
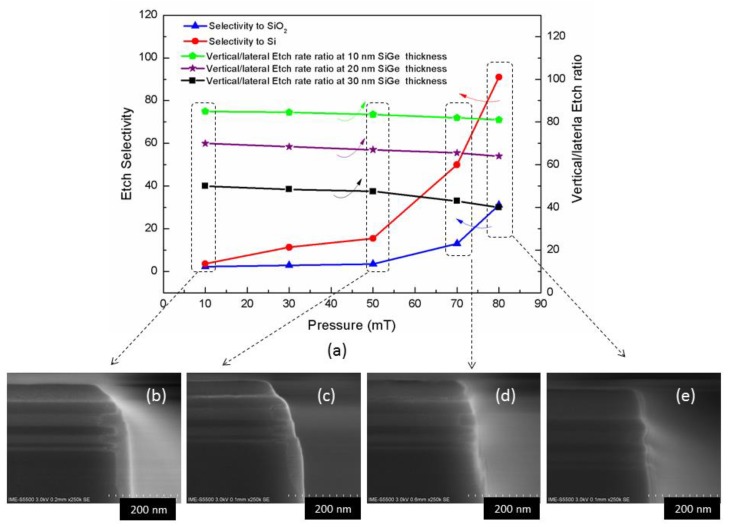
Effect of pressure on etching: (**a**) the dependence of etch selectivity and vertical/lateral etch ratio on pressure; (**b**) etching profile of 10mT; (**c**) etching profile of 50 mT; (**d**) etching profile of 70 mT; (**e**) etching profile of 80 mT.

It is well known that the process pressure is an important parameter for plasma etching, because it greatly affects the average free path and energy of ions. In this study the influence of pressure on the etch profile has been investigated according to parameters: *x* mTorr/source RF 250 W/bias RF 35 W/20 sccm CH_4_/25 sccm CH_2_F_2_/20 sccm O_2_/50 sccm Ar. It can be seen from Figure 7a that the etching selection ratio has been increasing along with the increasing pressure, especially over 50 mT. This mechanism can be explained by increasing the pressure and reducing the bombardment energy of the ions [35]. The anisotropy is slightly reduced, but the change is relatively small, which also helps to completely etch the silicon nitride on the outside of the sidewall. From Figure 7b–e, it can be seen that the amount of silicon nitride remaining on the sidewall gradually decreases until Figure 7e which shows no obvious residue (more detailed characterization will be performed at 3.5), and the remaining hard mask is getting thicker and thicker, indicating that the selectivity becomes getting higher. It should be noted that, due to the limitation of the vacuum gauge of the etcher tool (TCP 9400 DFM), the full-scale pressure can only be tested to 80mT, therefore, whether higher pressure has better results remains to be studied in the future.

In order to provide a larger insight of our results, a comparison was made with previously published references as shown in Table 1. The etching selectivity ratio in this study have some advantages over conventional etching results. Comparing with remote plasma and QALE, the selectivity to SiO_2_ is lower, but it has obvious advantages in etching anisotropy, which is crucial to control the accuracy of the final thickness of inner spacer.

### 3.5. Material Quality and Interface Analysis 

**Figure 8:**
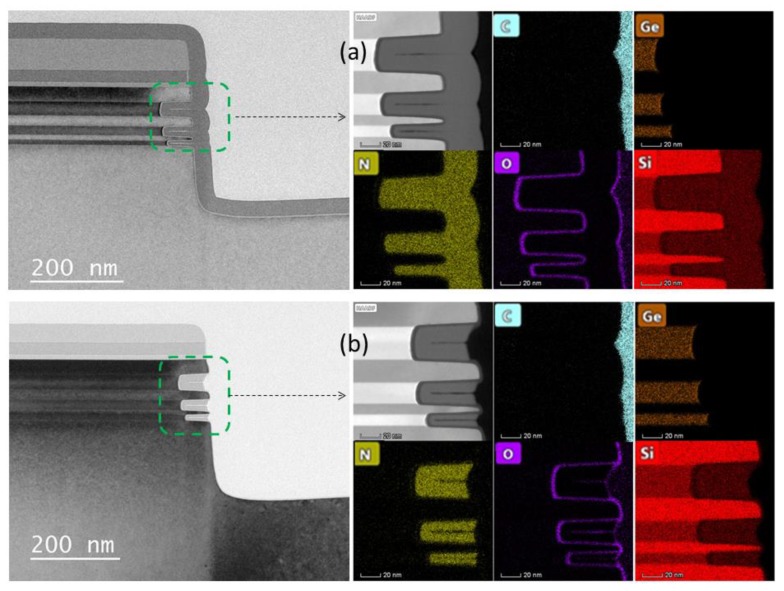
TEM and EDS micrographs: (**a**) LPCVD Silicon nitride inner spacer deposition; (**b**) inner spacer after etching under optimal conditions.

In order to more accurately characterize the results of the relatively optimal processes in this study, TEM and EDS characterizations were performed on the relatively optimal conditions of LPCVD filling and etching inner spacers. The outcome is shown in Figure 8. The figure shows that the silicon nitride film fills the Si/SiGe stack cavity in the sidewall very well, and only a small gap is found in the high-resolution TEM picture. These small gaps do not affect device integration and performance, but on the contrary, these gaps will improve device performance as it will further reduce parasitic capacitance [36]. 

The EDS mapping results show the distribution of silicon nitride film. The Si, Ge and O elements are basically consistent with the expected design results, there is no Ge diffusion during the LPCVD process. The C element is mainly from the TEM sample preparation, because it uses a carrier containing C and can only be referenced. It can be seen from Figure 8b that after the inner spacer is etched, except for the silicon nitride in the Si/SiGe stack cavity, the silicon nitride in the other positions have been etched completely. In particular, the end of the Si nanosheet is free of N elements (there is no silicon nitride residues), and only a thin layer of SiO_2_ is formed. Subsequently, this silicon oxide can be further removed during the growth of the epitaxial source and drain.

## 4. Conclusions

Inner spacer for GAA nano-structure, LPCVD silicon nitride has significantly better cavity filling effect than PECVD. The conventional ICP etching tool and the optimized CH_2_F_2_/O_2_/CH_4_/Ar gas mixtures can control the silicon nitride inner spacer etching effect very well. The ratio of silicon nitride etch selectivity to Si is more than 100:1, and that for the selectivity to SiO_2_ is more than 30:1. The vertical/lateral etch ratio is related to the thickness of SiGe, that is, the thinner the thickness of SiGe is, the higher the ratio is. For the nano-structure with a SiGe thickness of 10 nm, the vertical/lateral etching ratio reaches 80:1. The high-resolution TEM and EDS mapping results show that the SiN on the end face of the nanosheet is totally etched while the SiN in the cavity remains relatively intact. This method proposed in this study has the advantages of simple hardware equipment, high etching selectivity and excellent vertical/ lateral etching ratio. 

## Figures and Tables

**Table 1:** Selectivity of SiN etch to Si and SiO_2_, vertical/lateral etch ratio and etch accuracy for SiGe/Si inner spacer structure.

Parameter	Data in This Work ^1^	Ref. [21] ^2^	Ref. [22] ^3^	Ref. [24] ^4^
Selectivity to Si	101.5	6.2	100	-- ^5^
Selectivity to SiO_2_	31.6	18.6	70	100
Vertical/lateral etch ratio	82.5	-- ^5^	1	8
Etch accuracy (%)	2	-- ^5^	-- ^5^	-- ^5^

^1^ Pressure 80 mTorr/source RF 250 W/bias RF 35 W/20 sccm CH_4_/25 sccm CH_2_F_2_/20 sccm O_2_/50 sccm Ar. ^2^ Data of typical conventional plasma methods. ^3^ Data of special method—typical remote downstream plasma. ^4^ Data of special method—typical quasi-atomic layer etching. ^5^ Related data are unknown

## References

[1] Saremi M., Afzali-Kusha A., Mohammadi S. (2012). Ground plane fin-shaped field effect transistor (GP-FinFET): A FinFET for low leakage power circuits. Microelectron. Eng..

[2] Imenabadi R.M., Saremi M., Vandenberghe W. (2017). A Novel PNPN-Like Z-Shaped Tunnel Field- Effect Transistor With Improved Ambipolar Behavior and RF Performance. IEEE Trans. Electron Devices.

[3] Abadi R.M.I., Saremi M. (2018). A Resonant Tunneling Nanowire Field Effect Transistor with Physical Contractions: A Negative Differential Resistance Device for Low Power Very Large Scale Integration Applications. J. Electron. Mater..

[4] Radamson H., He X., Zhang Q., Liu J., Cui H., Xiang J., Kong Z., Xiong W., Li J., Gao J. (2019). Miniaturization of CMOS. Micromachines.

[5] Radamson H.H., Zhang Y., He X. (2017). The Challenges of Advanced CMOS Process from 2D to 3D. Appl. Sci.-Basel.

[6] Moon D.-I., Choi S.-J., Duarte J.P., Choi Y.-K. (2013). Investigation of Silicon Nanowire Gate-All-Around Junctionless Transistors Built on a Bulk Substrate. IEEE Trans. Electron Devices.

[7] Zhang Q., Yin H., Meng L., Yao J., Li J., Wang G., Li Y., Wu Z., Xiong W., Yang H. (2018). Novel GAA Si Nanowire p-MOSFETs With Excellent Short-Channel Effect Immunity via an Advanced Forming Process. IEEE Electron Device Lett..

[8] Mertens H., Ritzenthaler R., Chasin R. Vertically Stacked Gate-All-Around Si Nanowire CMOS Transistors with Dual Work Function Metal Gates. Proceedings of the IEEE 2016 IEEE International Electron Devices Meeting (IEDM).

[9] Yin X., Zhang Y., Zhu H., Wang G.L., Li J.J., Du A.Y., Xie L. (2020). Vertical Sandwich Gate-All-Around Field-Effect Transistors with Self-Aligned High-k Metal Gates and Small Effective-Gate-Length Variation. IEEE Electron Device Lett..

[10] Loubet N., Hook T., Montanini P. Stacked Nanosheet Gate-All-Around Transistor to Enable Scaling Beyond FinFET. Proceedings of the IEEE 2017 Symposium on VLSI Technology.

[11] Mertens H., Ritzenthaler R., Pena1 V. Vertically Stacked Gate-All-Around Si Nanowire Transistors:Key Process Optimizations and Ring Oscillator Demonstration. Proceedings of the IEEE 2017 IEEE International Electron Devices Meeting (IEDM).

[12] Kal S., Pereira C., Oniki Y. Selective isotropic etching of Group IV semiconductors to enable gate all around device architectures. Proceedings of the 19th The Surface Preparation and Cleaning Conference (SPCC).

[13] Oniki Y., Altamirano-Sánchez E., Holsteyns F. (2019). Selective Etches for Gate-All-Around (GAA) Device Integration: Opportunities and Challenges. ECS Trans..

[14] Loubet N., Kal S., Alix C. A Novel Dry Selective Etch of SiGe for the Enablement of High Performance Logic Stacked Gate-All-Around NanoSheet Devices. Proceedings of the IEEE 2019 IEEE International Electron Devices Meeting (IEDM).

[15] Li J., Wang W., Li Y., Zhou N., Wang G., Kong Z., Fu J., Yin X., Li C., Wang X. (2020). Study of selective isotropic etching Si_1−x_Ge_x_ in process of nanowire transistors. J. Mater. Sci. Mater. Electron..

[16] Li J., Li Y., Zhou N., Wang G., Zhang Q. (2020). A Novel Dry Selective Isotropic Atomic Layer Etching of SiGe for Manufacturing Vertical Nanowire Array with Diameter Less than 20 nm. Materials.

[17] Koehler F.H., Triyoso D., Hussain I. (2014). Challenges in spacer process developmen for leading-edge high-k metal gate technology. Phys. Status Solidi-R..

[18] Hållstedt J., Hellström P., Radamson H.H. (2008). Sidewall transfer lithography for reliable fabrication of nanowires and deca-nanometer MOSFETs. Thin Solid Films.

[19] Kaneko A., Yagishita A.K., Yahashi T. Sidewall Transfer Process and Selective Gate Sidewall Spacer Formation Technology for Sub-15nm FinFET with Elevated Source/Drain Extension. Proceedings of the IEEE 2005 IEEE International Electron Devices Meeting (IEDM).

[20] Kastenmeier B., Matsuo P., Beulens J. (1996). Chemical dry etching of silicon nitride and silicon dioxide using CF_4_/O_2_/N_2_ gas mixtures. J. Vac. Sci. Technol. A.

[21] Nakayama D., Wada A., Kubota1 T. (2013). Highly selective silicon nitride etching to Si and SiO2 for a gate sidewall spacer using a CF_3_I/O_2_/H_2_ neutral beam. J. Phys. D Appl. Phys..

[22] Kastenmeier B.E.E., Matsuo P.J., Oehrlein G.S. (1999). Highly selective etching of silicon nitride over silicon and silicon dioxide. J. Vac. Sci. Technol. A.

[23] Sherpa S.D., Ranjan A. (2017). Quasi-atomic layer etching of silicon nitride. J. Vac. Sci. Technol. A.

[24] Posseme N., Ah-Leung V., Pollet O. (2016). Thin layer etching of silicon nitride: A comprehensive study of selective removal using NH_3_/NF_3_ remote plasma. J. Vac. Sci. Technol. A.

[25] Posseme N., Pollet O., Barnola S. (2014). Alternative process for thin layer etching: Application to nitride spacer etching stopping on silicon germanium. Appl. Phys. Lett..

[26] Radamson H.H., Simoen E., Luo J., Zhao C. (2018). Past, Present and Future of CMOS.

[27] Radamson H.H., Thylen L. (2014). Monolithic Nanoscale Photonics-Electronics Integration in Silicon and Other Group 1V Elements.

[28] Wang G., Abedin A., Moeen M., Kolahdouz M., Luo J., Guo Y., Zhao C. (2018). Integration of highly-strained SiGe materials in 14 nm and beyond nodes FinFETtechnology. Solid State Electron..

[29] Wang G., Luo J., Qin C., Liang R., Xu Y., Liu J., Xu J. (2017). Integration of Highly Strained SiGe in Source and Drain with HK and MG for 22 nm Bulk PMOS Transistors. Nanoscale Res. Lett..

[30] Zhang Q., Tu H., Gu S. (2018). Influence of Rapid Thermal Annealing on Ge-Si Interdiffusion in Epitaxial Multilayer Ge_0.3_Si_0.7_/Si Superlattices with Various GeSi Thicknesses. ECS J. Solid State Sci. Technol..

[31] Bangsaruntip S., Cohen G.M., Majumdar A., Zhang Y., Engelmann S.U., Fuller N.C.M., Gignac L.M., Mittal S., Newbury J.S., Guillorn M. High performance and highly uniform gate-all-around silicon nanowire MOSFETs with wire size dependent scaling. Proceedings of the IEEE 2009 IEEE International Electron Devices Meeting (IEDM).

[32] Bangsaruntip S., Balakrishnan K., Cheng S.-L. Density scaling with gate-all-around silicon nanowire MOSFETs for the 10 nm node and beyond. Proceedings of the IEEE 2013 IEEE International Electron Devices Meeting (IEDM).

[33] Nishimura H., Takagi S., Fujino M., Nishi N. (2002). Gap-Fill Process of Shallow Trench Isolation for 0.13 µm Technologies. Jpn. J. Appl. Phys..

[34] Nishimura H., Takagi S., Joshi M., Eranna G., Runthala D. (2000). LPCVD and PECVD silicon nitride for microelectronics technology. Indian J. Eng. Mater. Sci..

[35] Donnelly V.M., Kornblit A. (2013). Plasma etching: Yesterday, today, and tomorrow. J. Vac. Sci. Technol. A.

[36] Park J., Hu C. Air Spacer MOSFET Technology for 20nm Node and Beyond. Proceedings of the IEEE 2008 9th International Conference on Solid-State and Integrated-Circuit Technology (ICSICT).

